# Approaches to ascertaining comorbidity information: validation of routine hospital episode data with clinician-based case note review

**DOI:** 10.1186/1756-0500-7-253

**Published:** 2014-04-21

**Authors:** Martin Soo, Lynn M Robertson, Tariq Ali, Laura E Clark, Nicholas Fluck, Marjorie Johnston, Angharad Marks, Gordon J Prescott, William Cairns S Smith, Corri Black

**Affiliations:** 1 Aberdeen Applied Renal Research Collaboration, Division of Applied Health Sciences, University of Aberdeen, Polwarth Building, Foresterhill, AB25 2ZD Aberdeen, Scotland; 2 NHS Grampian, Aberdeen, Scotland; 3 King Faisal Specialist Hospital Research Centre, Riyadh, Saudi Arabia

**Keywords:** Chronic kidney disease, Validation study, Medical record linkage, Patient outcomes, Public health

## Abstract

**Background:**

In clinical practice, research, and increasingly health surveillance, planning and costing, there is a need for high quality information to determine comorbidity information about patients. Electronic, routinely collected healthcare data is capturing increasing amounts of clinical information as part of routine care. The aim of this study was to assess the validity of routine hospital administrative data to determine comorbidity, as compared with clinician-based case note review, in a large cohort of patients with chronic kidney disease.

**Methods:**

A validation study using record linkage. Routine hospital administrative data were compared with clinician-based case note review comorbidity data in a cohort of 3219 patients with chronic kidney disease. To assess agreement, we calculated prevalence, kappa statistic, sensitivity, specificity, positive predictive value and negative predictive value. Subgroup analyses were also performed.

**Results:**

Median age at index date was 76.3 years, 44% were male, 67% had stage 3 chronic kidney disease and 31% had at least three comorbidities. For most comorbidities, we found a higher prevalence recorded from case notes compared with administrative data. The best agreement was found for cerebrovascular disease (κ = 0.80) ischaemic heart disease (κ = 0.63) and diabetes (κ = 0.65). Hypertension, peripheral vascular disease and dementia showed only fair agreement (κ = 0.28, 0.39, 0.38 respectively) and smoking status was found to be poorly recorded in administrative data. The patterns of prevalence across subgroups were as expected and for most comorbidities, agreement between case note and administrative data was similar. Agreement was less, however, in older ages and for those with three or more comorbidities for some conditions.

**Conclusions:**

This study demonstrates that hospital administrative comorbidity data compared moderately well with case note review data for cerebrovascular disease, ischaemic heart disease and diabetes, however there was significant under-recording of some other comorbid conditions, and particularly common risk factors.

## Background

The importance of electronic, routinely collected health care information has been at the forefront of discussion in recent years. Substantial investment by healthcare providers internationally in digital health systems is capturing increasing amounts of clinical information as part of routine care [1-3]. The potential application of such data extends beyond the ‘day to day care’ of individual patients; with important roles in planning and costing health services, population health surveillance and research.

In the UK, information about an episode of hospital care is recorded following a patient’s discharge. Details of diagnoses are coded using the World Health Organisation’s International Classification of Disease (ICD) [4]. In Scotland, this information is recorded on the Scottish Morbidity Record (SMR01), which is collated nationally by the Information Services Division (ISD), part of NHS National Services Scotland, and data have been routinely available since 1980. The accuracy of such data is important to a wide range of users. Changes in coding practice, administration systems and the increasing complexity of patients’ health care records, driven by increasing life expectancy, and the growing burden of chronic disease, may all impact on the quality of recorded data. Quality assurance assessment of the recording of clinical codes for diagnoses associated with individual episodes of hospitalisation for Scottish hospital episode data in 2010–11, has shown high accuracy (88% for the Main Condition and 82% for Other Conditions) [5].

Comorbidity describes the burden of illness co-existing with a particular disease of interest which may impact on patient outcomes. Comorbidity is an important dimension in health care that is under-reported and under-investigated due to methodological challenges in its assessment. In clinical practice, research, and increasingly health surveillance, planning and costing, there is a need for high quality information to determine comorbidity information about patients. Here, rather than a single hospital episode being reviewed, longer periods of data might be examined for evidence of comorbid conditions. Traditionally, clinician-based case note review (CNR) has been regarded as the ‘gold’ standard method of extracting comorbidity information. However, CNR is labour- and resource-intensive. Electronic, routinely collected healthcare data offer a potentially important alternative approach [6,7].

A systematic review published in 2009 [8] reported that routine administrative data had limited validity for comorbidity assessment. However, the studies were often small, included only selected diagnoses, and none were from the UK. Recent studies assessing routinely collected comorbidity data in cohorts of patients with disease demonstrate the variability in results, reporting kappa coefficients of 0.67 to 0.93 [9] and 0.32 to 0.75 [10]. In addition, recent updates have shown a slight improvement in coding of comorbidity over time when looking at the validity of single episode coding of comorbidity [11].

Patients with chronic kidney disease (CKD) are often elderly and the presence of comorbidity is common; the cohort used in this study provides a useful model for understanding the recording of comorbidity in routine administrative data as compared to clinician-based CNR, particularly in those with a chronic disease. Here we aim to present a validation study of hospital episode data (with five years look-back) compared with clinician-based CNR as a means of identifying comorbidity in a CKD cohort in the UK.

## Methods

### Study design

We undertook a validation study using record linkage. An established population based clinical cohort for CKD, the Grampian Laboratory Outcomes Mortality and Morbidity Study-1 (GLOMMS-I) was linked to a routinely collected hospital administrative dataset.

### GLOMMS-I

The GLOMMS-I cohort, which comprised 3426 patients with moderate to severe CKD identified from the general population, is described elsewhere [12]. GLOMMS-I participants were identified from screening of all routine laboratory biochemistry data collected from hospital and primary care for a population of 433,109 adults (>15 years of age) representing a single health administrative region in the North East of Scotland between 1 January and 30 June 2003. Individuals were included in GLOMMS-I if they met the Kidney Disease Outcomes Quality Initiative (KDOQI) definition of stage 3 to 5 CKD [13] (glomerular filtration rate (GFR) < 60 mL/min/1.73 m^2^ for at least three months). The date of the first estimated GFR (eGFR) <60 mL/min/1.73 m^2^ during the period January to June 2003 was taken as the ‘index’ date for each patient.

### Case note review and administrative data

For this validation study, data were derived from the GLOMMS-I cohort and linked with a hospital administrative dataset that recorded discharge diagnoses for all hospitalisations in the region (SMR01). In GLOMMS-I, CNR had been undertaken to establish baseline comorbidity. Clinical information had been extracted from patients’ hospital medical records by two physicians, experienced in nephrology and general medicine, and blinded to the SMR01 data. Information was recorded on a standardised form. Data were entered by a data co-ordinator and a 10% sample checked for accuracy by an independent assessor. Data were collected on selected comorbidities (cerebrovascular disease, peripheral vascular disease, congestive cardiac failure, types I and II diabetes mellitus, dementia, chronic obstructive pulmonary disease, connective tissue diseases, haematological malignancy, non-haematological malignancy, chronic liver disease and smoking status) present at any time prior to, but not including any admissions at the time of the index blood sample. Data were also collected on ischaemic heart disease and hypertension, however these events were recorded up to one year post-index.

In Scotland, information about an episode of hospital care is recorded on the SMR01, and diagnoses are classified according to ICD-10 [4]. All relevant diagnoses and procedures identified and recorded by medical personnel during admission are, following discharge, then coded by trained professional coders using appropriate documentation, which may include discharge summaries and/or medical records. Comorbidities thought to be important to outcome in CKD and that contribute to Charlson were included. Codes were identified for these comorbidities from the ICD-10 manual (Table 1). SMR01 data were obtained for all diagnoses, except for ischaemic heart disease and hypertension, for the five years prior to the index date, *excluding* admission at index date. For ischaemic heart disease and hypertension, SMR01 data for the year 2003 were included to match CNR time periods.

**Table 1:** ICD-10 codes for diagnoses

**Diagnosis**	**ICD-10 Code (2007 version)**
Ischaemic heart disease	I20 to I25
Hypertension	I10 to I15, I67.4, I70.1, O10, O11
Cerebrovascular disease	I60.x-I69.x, G45.x, G46.x, H34.0, I63, I64, I69.3, I69.4
Peripheral vascular disease	I70.x, I71.x, I73.1, I73.8, I73.9, I77.1, I79.0, I79.2
Congestive cardiac failure	I50.x, I11.0, I13.0, I13.2, I25.5, I42.0, I42.5–I42.9, I43.x
Diabetes mellitus	E10, O24.0, E11, O24.1, E14, E13, E12
Dementia	F01, F00, F03, F02, F05.1, G30, G31.1
Chronic obstructive pulmonary disease	I27.8, I27.9, J40-J47, J60-J67, J68.4, J70.1, J70.3
Connective tissue disease	M05, M06, M07, M08, M30, M31, M32, M33, M34, M35, M45
Haematological malignancy	C81 to C96
Non-haematological malignancy	C00 to C75, C76 to C80, C97
Chronic liver disease	B18, K70.0, K70.2-70.9, K70.0-70.3, K71.3-71.5, K71.7, K71.1, K72.9, K76.5, K72.1, K73, K74, K76.6, K76.7, K76.0, K76.2-76.4, K76.8, K76.9, Z94.4, I85.0, I85.9, I86.4, I98.2
Smoking status	Z72.0, F17.2, Z71.6

SMR01 and CNR definitions of included comorbidities are available in Additional file 1. A measure of rurality (Scottish Government 6-fold Urban Rural Classification [14]) and socioeconomic status (Scottish Index of Multiple Deprivation (SIMD) 2009 quintiles) [15] were also obtained through linkage of patient postcode at baseline with administrative data.

### Data linkage

SMR01 data for the selected comorbidities were provided by ISD [16]. The Community Health Index (CHI) number, a unique patient identifier used throughout the Scottish health care system, was used to link GLOMMS-I patients with their SMR01 data using deterministic matching. Patient identifiers were removed after data linkage. The dataset was stored in the Grampian Data Safe Haven allowing secure controlled access for researchers while ensuring data security [17]. Because of inconsistencies in the CHI number and other data, 206 individuals from GLOMMS-I did not have both CNR and SMR01 data, and were excluded. There was one duplicate record which was also excluded. Overall, 3219 patients were included in this study.

### Statistical analysis

Descriptive analyses were performed reporting counts and percentages. To assess agreement between CNR and SMR01 recorded comorbidity (with CNR serving as the reference), prevalence, kappa statistic, sensitivity, specificity, positive predictive value and negative predictive value were calculated for each comorbidity, and 95% confidence intervals (CI) calculated using the Wilson method [18]. The kappa statistic is a measure of agreement between two sets of categorical measurements on the same individuals, first categorised by Landis and Koch [19]. We categorised agreement as poor if κ ≤ 0.20, fair if 0.21 ≤ κ ≤ 0.40, moderate if 0.41 ≤ κ ≤0.60, substantial if 0.61 ≤ κ ≤ 0.80 and good if κ > 0.80 [20]. All analyses were performed using STATA 12.1 and Microsoft Excel. For subgroup analysis, the study population was categorised by age group (<75 yrs and ≥75 yrs), sex, CKD stage (stage 3 and stages 4/5), presence of comorbidities (<3 and ≥3, excluding smoking status), with or without a diagnosis of ischaemic heart disease or malignancy, the Scottish Government 6-fold Urban Rural Classification (1/2 (urban) and 3–6 (more rural) and SIMD quintiles (1–3 (more deprived) and 4/5 (less deprived)). CKD stage was assigned using the index eGFR.

This study was approved as part of GLOMMS-I, by the University of Aberdeen Research Ethics Committee and the NHS Grampian Caldicott Guardian and discussed with the North of Scotland NHS Research Ethics Committee.

## Results

### Characteristics of study population

The baseline characteristics of the 3219 study participants are summarised in Table 2. The cohort represents a relatively elderly population, with a median age at index date of 76.3 years. Forty-four per cent were male, 67.0% had stage 3 CKD and 31.4% had at least three comorbidities.

**Table 2:** Characteristics of the study population

**Characteristic**	***n***	**%**
**Total**	**3219**	l00.0
**Sex**		
Male	1,414	43.9
Female	1,805	56.1
**Age at index**		
Median age (years)	76.3	
15-44	84	2.6
45-54	105	3.2
55-64	269	8.4
65-74	722	22.4
75-84	1,348	41.9
≥85	691	21.5
**CKD stage at index**		
3	2.156	67.0
4	973	30.2
5	90	2.8
Number of morbidities*^, **†**^		
**0**	315	9.8
1	896	27.8
2	1,002	31.1
3	643	20.0
4	272	85
5	73	2.3
6	16	0.5
7	2	0.1
Scottish government 6-fold urban rural classification*		
1 Large urban areas	1,266	39.3
2 Other urban areas	513	15.9
3 Accessible small towns	266	8.3
4 Remote small towns	299	9.3
5 Accessible rural areas	498	15.5
6 Remote rural areas	338	10.5
Missing	39	1.2
SIMD 2009 quintlle*		
1 most deprived	234	7.3
2	538	16.7
3	812	25.2
4	867	26.9
5 least deprived	729	22.7
Missing	39	1.2

SIMD, Scottish Index of Multiple Deprivation.

*based on case note review.

^†^excluding smoking status.

### Prevalence of comorbidities in GLOMMS-I

**Figure 1:**
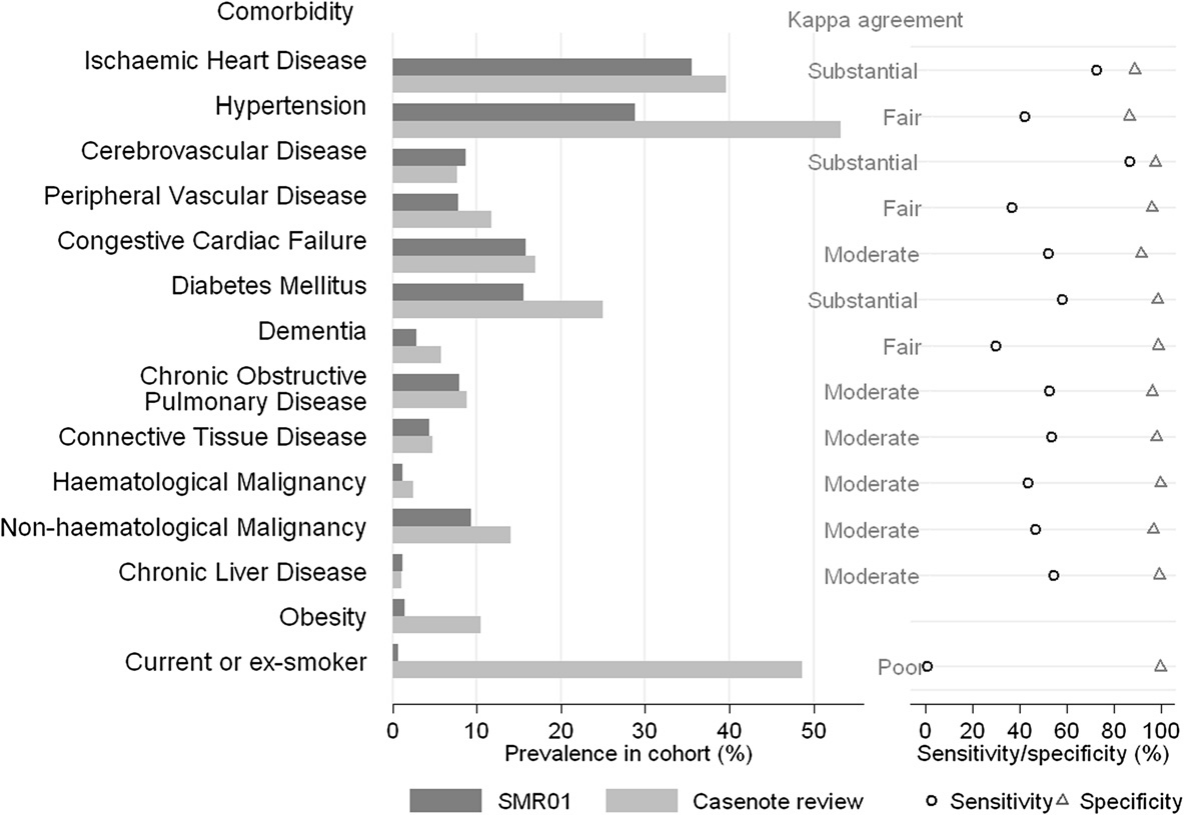
Prevalence, kappa agreement, sensitivity and specificity for SMR01 and case note review recorded comorbidity.

With the exception of cerebrovascular and chronic liver diseases, the estimated prevalence of all comorbidities was higher based on CNR compared with SMR01 (Figure 1 and Table 3), although differences were generally small. Ischaemic heart disease had the highest prevalence in SMR01 data at 35.6%, with similar CNR prevalence (39.7%). Hypertension had the highest prevalence in CNR data at 53.3%, however, hypertension was only recorded in 28.8% of the SMR01 data. Smoking status also showed a difference with 48.6% recorded from CNR as current or ex-smokers but only 0.7% in SMR01.

**Table 3:** Agreement between SMR01 and case note review recorded comorbidity

	**Prevalence**													
**Co-morbidity**	**SMRO1**		**CNR**		Kappa*		**Sensitivity**		**Specificity**		**PPV**		**NPV**	
	**n**	**%**	**n**	**%**	**Value**	**Label**	**%**	**95% CI**	**%**	**95% CI**	**%**	**95% CI**	**%**	**95% CI**
Ischaemic heart disease	1146	35.6	1277	39.7	0.63	Substantial	72.8	(70.3-75.2)	88.9	(87.4-90.2)	81.2	(78.8-83.3)	83.3	(81.6-84.8)
Hypertension	928	28.8	1715	53.3	0.28	Fair	42.4	(40.1-44.8)	86.7	(84.9-88.3)	78.4	(75.7-81.0)	56.9	(54.9-58.9)
Cerebrovascular disease	282	8.8	248	7.7	0.80	Substantial	87.1	(82.4-90.7)	97.8	(97.2-98.3)	76.6	(71.3-81.2)	98.9	(98.5-99.2)
Peripheral vascular	250	7.8	379	11.8	0.39	Fair	36.9	(32.2-41.9)	96.1	(95.4-96.8)	56.0	(49.8-62.0)	92.0	(90.9-92.9)
Congestive cardiac failure	511	15.9	546	17.0	0.45	Moderate	52.4	(48.2-56.5)	91.6	(90.5- 92.6)	56.0	(51.6-60.2)	90.4	(89.2-91.5)
Diabetes mellitus	501	15.6	805	25.0	0.65	Substantial	58.4	(54.9-61.7)	98.7	(98.2-99.1)	93.8	(91.4-95.6)	87.7	(86.4-88.9)
Dementia	91	2.8	187	5.8	0.38	Fair	29.9	(23.8-36.9)	98.8	(98.4-99.2)	61.5	(51.3-70.9)	95.8	(95.1-96.5)
Chronic obstructive pulmonary disease	255	7.9	283	8.8	0.51	Moderate	52.7	(46.8-58.4)	96.4	(95.7-97.0)	58.4	(52.3-64.3)	95.5	(94.7-96.2)
Connective tissue disease	140	4.3	154	4.8	0.54	Moderate	53.9	(46.0-61.6)	98.1	(97.6-98.6)	59.3	(51.0. 67.1)	97.7	(97.1-98.2)
Haematological malignancy	39	1.2	78	2.4	0.57	Moderate	43.6	(33.1 . 54.6)	99.8	(99.6-99.9)	87.2	(73.3-94.4)	98.6	(98.1-99.0)
Non-haematological malignancy	300	9.3	454	14.1	0.51	Moderate	47.1	(42.6-51.7)	96.9	(96.2-97.5)	71.3	(66.0-76.2)	91.8	(90.7-92.7)
Chronic liver disease	37	1.1	33	1.0	0.51	Moderate	54.5	(38.0-70.2)	99.4	(99.1-99.6)	48.6	(33.4-64.1)	99.5	(99.2-99.7)
Current or ex-smoker	21	0.7	1564	48.6	0.01	Poor	1.1	(0.7-1.7)	99.8	(99.4-99.9)	81.0	(60.0-92.3)	51.6	(49.9-53.4)

SMRO1. Scottish Morbidity Record 01; PPV, Positive Predictive Value; NPV, Negative Predictive Value; CI, Confidence Interval.

*Interpretation of kappa: Agreement poor if κ ≤ 0.20, fair if 0.21κ ≤ O.40, moderate if 0.41κ ≤ 0.60, substantial if 0.61 ≤ 0.80 and good if κ > 0.80.

### Agreement between CNR and SMR01 recorded comorbidities

Using CNR as the reference, kappa, sensitivity, specificity, positive predictive value and negative predictive value for each diagnosis are reported in Figure 1 and Table 3. For most comorbidities, kappa values were ≥0.41, indicating at least moderate agreement. Good agreement was found for cerebrovascular disease with a kappa value of 0.80. Ischaemic heart disease and diabetes had substantial agreement, with kappa values of 0.63 and 0.65 respectively. Hypertension, peripheral vascular disease and dementia showed only fair agreement (κ = 0.28, 0.39, 0.38 respectively). Smoking status showed poor agreement (κ = 0.01).

The sensitivity of SMR01 data generally reflected the kappa value. Peripheral vascular disease showed a sensitivity of 87%, whereas smoking status sensitivity was only 1.1%. This means that most people with peripheral vascular disease recorded from CNR were also identified by SMR01 whereas there were few smokers identified by SMR01 data. However, the specificity of the SMR01 data was generally very high, with all values over 85% and all but three conditions having a specificity >95%. The negative predictive value was generally >80% except for hypertension and smoking status. The positive predictive value ranged between 49% and 94%, with both diabetes and haematological malignancy being high, whereas chronic liver disease and peripheral vascular disease were low.

### Subgroup analysis

Results of the subgroup analysis are available in Additional file 2. For some comorbidities, numbers were small, and results should be interpreted with caution. Analysing males and females separately, the prevalence of vascular diseases (ischaemic heart disease, cerebrovascular disease, peripheral vascular disease and congestive cardiac failure) were all higher in males. The prevalence of dementia was higher in females. Agreement was similar for the majority of comorbidities, however for dementia the kappa value was higher in females compared to males.

The prevalence of comorbidities showed higher vascular diseases in those ≥75 years and higher rates of diabetes in those <75 years. For ischaemic heart disease, congestive cardiac failure, connective tissue disease, haematological malignancy and chronic liver disease, kappa values were higher for those <75 compared with those ≥75 years. The majority of comorbidities showed no substantial differences in kappa values comparing CKD stage 3 with CKD stages 4 and 5.

For most comorbidities, there was no substantial difference in agreement comparing those with <3 and ≥3 comorbidities. For peripheral vascular disease, congestive cardiac failure, chronic obstructive pulmonary disease and connective tissue disease, kappa values were higher in the group with ≥3 compared with <3 comorbidities.

Those with ischaemic heart disease had a higher prevalence of other vascular diseases. However, kappa values were similar for those with and without ischaemic heart disease. We also compared patients with and without a diagnosis of any malignancy in their case notes. The prevalence of other comorbid disease was lower in those with malignancy than those without, except chronic obstructive pulmonary disease and current or ex-smoking status. For most of the comorbidities, kappa values were similar.

Subgroup analysis for urban–rural classification showed a slight trend for increased prevalence of all comorbidities in those who lived in more urban areas. Comparing those most and least deprived, there appeared to be a higher prevalence of chronic obstructive pulmonary disease in those in more deprived areas. However, kappa values were similar for all comorbidities by urban–rural and SIMD groups.

## Discussion

In this large population study, we compared clinician-based case note review to routine hospital episode administrative data as methods of determining co-morbid status. We compared the recording of 13 major health conditions in a five year “look-back” period using administrative data and a clinician-based assessment of the paper medical record. Hospital data generally compared moderately well with CNR. The prevalence of most conditions was lower using the hospital administration data, and for most comorbidities, agreement was at least moderate. Similar findings have been reported by others [8-10].

To the best of our knowledge, this is the largest validation study of comorbidity in a population based cohort, and only the second comorbidity validation study in people with CKD. The only other CKD study only validated 184 records [21]. The study cohort mostly comprised of elderly patients with a chronic disease. Our findings may not relate to younger and healthier populations but, as a population based cohort, they are likely to reflect well the findings for people with chronic disease.

Agreement was only fair for hypertension, peripheral vascular disease and dementia and it was poor for smoking status. For hypertension, the specificity was high, but only 42% of patients observed as hypertensive on CNR had hypertension noted in their administrative data. This is not unexpected. While hypertension is an important risk factor for other conditions, it is generally managed in the outpatient setting, and, with modern therapies, is uncommonly a major reason for hospital admission. However, this is not a consistent finding across all studies [9,10,21].

Smoking, the single most important risk factor for many common chronic diseases, was barely recorded in the hospital administration data, despite a high level of recording in the case notes, and a high prevalence of current smokers. This again reflects that smoking is rarely the main reason for admission and is therefore, not recorded by hospital administration data on discharge. The very low recording in hospital administration data of factors such as hypertension and smoking limits the utility of such data for research of these important health risks. It also has major implications for the utility of the data for health surveillance and service planning. Yet, CNR demonstrates that the information was recorded in clinical records and, with relatively minor changes to recording procedures, this information could be captured for future use or obtained from linkage to primary care records where such information regarding risk factors may be more complete.

To our knowledge, no other published studies have explored agreement between CNR and hospital episode data methods of recording comorbidity in clinically important subgroups. The patterns of prevalence across subgroups were as expected. For most comorbidities agreement between CNR and administrative data was similar across subgroups. Agreement was less, however, in older ages compared with data for those aged less than 75 years for some important comorbidities. In patients with three or more comorbidities, there was some evidence that administrative data performed better than for those without the additional comorbid burden. This may reflect regular contact with the health service resulting in more opportunities to code other diagnoses in the administrative data, or that for patients with complex healthcare needs and resulting large paper based case records, case note review becomes more challenging.

In this study, CNR data extraction was carried out by two physicians experienced in nephrology and general medicine. The physicians extracted information from different case notes and there was no test of inter-rater reliability. However, the data extraction was carried out prior to linkage with SMR01 data, thus minimising measurement bias. In addition, data entry was checked by an independent assessor. The cohort was identified from electronic laboratory records rather than recruited from a clinical setting and did not, therefore, have issues of participation bias. Our patients were predominantly of northern European Caucasian ethnicity and our findings may not be representative of other ethnic groups. There were a number of methodological challenges, largely relating to the nature of the study, which was not specifically designed for the purpose of validation of comorbidity recording methods. There was a difference in the time periods for the extraction of the administrative data and CNR data. The SMR01 data were extracted for the period five years prior to the index date whereas CNR recorded comorbidities at any time prior to the index date. A diagnosis may be identified in CNR but not in SMR01 data if there were no admissions in the five years prior to the index date. However, the length of look back period has been studied elsewhere [10,22,23]. Overall, longer look-back periods were better at identifying those with chronic disease.

This study demonstrates that routinely collected hospital administrative data can reasonably be used to determine a profile of a patient’s comorbidity from across their health records for the majority of conditions. Scottish hospital episode data recording of individual hospital events, recorded as part of service administration on hospital discharge, is of high quality and similar completeness as that of the rest of the UK [24]. The type of administrative data assessed in this study could be generalizable to similar systems where diagnoses are captured as part of administrative processes rather than “at the bedside” as part of the clinical record. The Scottish administrative data system was less able to detect risk factors such as smoking status and hypertension, which are less likely to be coded from the hospital record. As we increasingly move towards an electronic patient record, it will be important that there are continued efforts to code hospital events systematically to provide vital summary information relating to both the primary cause of admission and the associated significant comorbidity information. The widespread approach of making full text communication/records available, while a vital component of the electronic patient record, is not sufficient alone. The increasing use of coded recording in outpatient settings and the ability to link to primary care records will substantially improve the completeness of comorbidity recording, capturing conditions such as hypertension and risk factors such as smoking that are generally monitored in primary care. In the UK, payment by results approaches such as the Quality and Outcomes Framework have improved the regular recording of key risk factor information [25].

## Conclusion

The use of administrative healthcare data is increasingly important for understanding health and health care in research, health surveillance and healthcare planning, as recognised by the UK Department of Health [26]. This study demonstrates that hospital administrative comorbidity data generally compared moderately well with case note review data for cerebrovascular disease, ischaemic heart disease and diabetes, however there was significant under-recording of some other comorbid conditions and risk factors. Knowledge of the strengths and limitations of data are crucial for researchers and planners when interpreting findings based on administrative healthcare data.

## Competing interests

The authors declare that they have no competing interests.

## Authors’ contributions

CB, AM, NF, GP and WS conceived of the study. All authors participated in the design of the study. TA and LC undertook data acquisition for the case note review. MS, LR and AM carried out the data analysis. MS, LR, AM, MJ and CB drafted the manuscript. All authors participated in the interpretation of the data and read and approved the final manuscript.

## Supplementary Material

Additional file 1 Definitions of diagnoses: ICD-10 and case note review. Click here for file

Additional file 2 Results of subgroup analysis. Click here for file
