# Supplementary material for: Approaches to ascertaining comorbidity information: validation of routine hospital episode data with clinician-based case note review
Source: BMC Res Notes. 2014 Apr 21;7:253. doi: 10.1186/1756-0500-7-253 (PMC4022331; doi:10.1186/1756-0500-7-253)
Supplement: Additional file 1 — Definitions of diagnoses: ICD-10 and case note review. [file 1756-0500-7-253-S1.pdf]

Additional file 1: Definitions of diagnoses: ICD-10 and case note review

Diagnosis	SMR01 Definition		Case note review definition
	ICD-10 Code (2007 Version)	ICD-10 Definition	
Ischaemic Heart Disease	I20 to I25	Ischaemic Heart Diseases	Patients were considered to have ischaemic heart disease if there was evidence from the case notes or laboratory evidence of myocardial infarction or acute coronary syndrome at any time prior to the index creatinine and up to one year post-index. They were also considered to have ischaemic heart disease if their GP or other physician had recorded this diagnosis based on clinical, electrocardiography or angiography findings.
Hypertension	I10 to I15<br>I67.4<br>I70.1<br>O10<br><br>O11	Hypertensive diseases<br>Hypertensive encephalopathy<br>Atherosclerosis of renal artery<br>Pre-existing hypertension complicating pregnancy, childbirth and the puerperium<br>Pre-existing hypertension with superimposed proteinuria	Patients were considered to be hypertensive if receiving medication for hypertension and diagnosed to be hypertensive by the GP or while in hospital at any time prior to the index and up to one year post-index.
Cerebrovascular Disease	I60.x-I69.x<br>G45.x<br><br>G46.x<br><br>H34.0<br>I63<br>I64<br><br>I69.3<br>I69.4	Cerebrovascular disease<br>Transient cerebral ischaemic attacks and related syndromes<br>Vascular syndromes of the brain in cerebrovascular diseases<br>Transient retinal artery occlusion<br>Cerebral infarction<br>Stroke, not specified as haemorrhage or infarction<br>Sequelae of cerebral infarction<br>Sequelae of stroke	Patients were defined as having cerebrovascular disease if they had a history of transient ischaemic attack or previous cerebrovascular accident with full recovery or residual deficit. They were also classified as having cerebrovascular disease if they had an abnormal CT brain scan suggestive of vascular disease.
Peripheral Vascular Disease	I70.x<br>I71.x<br>I73.1<br>I73.8<br>I73.9	Atherosclerosis<br>Aortic aneurysm and dissection<br>Thromboangitis obliterans (Buerger)<br>Other specified peripheral vascular disease<br>Peripheral vascular disease, unspecified	Patients were classified as having peripheral vascular disease if they had a history of claudication or abnormal peripheral arteriogram or a history of tissue loss secondary to vascular disease.

Diagnosis	SMR01 Definition		Case note review definition
	**ICD-10 Code (2007 Version)**	**ICD-10 Definition**	
	I77.1<br>I79.0<br><br>I79.2	Stricture of artery<br>Aneurysm of the aorta in diseases classified elsewhere<br>Peripheral angiopathy in diseases classified elsewhere	
Congestive Cardiac Failure	I50.x<br>I11.0<br><br>I13.0<br><br>I13.2<br><br>I25.5<br>I42.0<br>I42.5–I42.9<br>I43.x	Heart failure<br>Hypertensive heart disease with congestive heart failure<br>Hypertensive heart and renal disease with congestive heart failure<br>Hypertensive heart and renal disease with both congestive heart failure and renal failure<br>Ischaemic cardiomyopathy<br>Dilated cardiomyopathy<br>Various cardiomyopathies<br>Cardiomyopathy in diseases specified elsewhere	Patients were classed as having congestive cardiac failure if clinical or radiological findings supported this or if it was diagnosed by a physician or GP.
Diabetes Mellitus	E10<br>O24.0<br><br>E11<br>O24.1<br><br>E14<br>E13<br>E12	Insulin dependent diabetes mellitus<br>DM in pregnancy: pre-existing DM, insulin-dependent<br>Non-insulin dependent diabetes mellitus<br>DM in pregnancy: pre-existing DM, non-insulin dependent<br>Unspecified diabetes mellitus<br>Other specified Diabetes Mellitus<br>Mal-nutrition related Diabetes Mellitus	Diabetes Type I and II recorded in case notes
Dementia	F01<br>F00<br>F03<br>F02<br>F05.1	Vascular dementia<br>Dementia in Alzheimers disease<br>Unspecified dementia<br>Dementia in other diseases specified elsewhere<br>Delirium superimposed on dementia	These were patients who had a history of dementia or a diagnosis made by a hospital physician or GP.

Diagnosis	SMR01 Definition		Case note review definition
	**ICD-10 Code (2007 Version)**	**ICD-10 Definition**	
	G30<br>G31.1	Alzheimers<br>Senile degeneration of the brain not elsewhere classified	
Chronic Obstructive Pulmonary Disease	I27.8<br>I27.9<br>J40-J47<br>J60-J67<br>J68.4<br><br>J70.1<br><br>J70.3	Other specified pulmonary heart diseases<br>Pulmonary heart disease unspecified<br>Chronic lower respiratory diseases<br>Lung diseases due to external agents<br>Chronic respiratory conditions due to chemicals, gases, fumes and vapours<br>Chronic and other pulmonary manifestations due to radiation<br>Chronic drug-induced interstitial lung disorders	Those who were diagnosed with chronic obstructive pulmonary disease by a respiratory physician or diagnosed by another physician with evidence from pulmonary function tests.
Connective Tissue Disease	M05<br>M06<br>M07<br>M08<br>M30<br>M31<br>M32<br>M33<br>M34<br>M35<br>M45	Seropositive Rheumatoid Arthritis<br>Other Rheumatoid arthritis<br>Psoriatic and enteropathic arthropathies<br>Juvenile arthritis<br>Polyarteritis nodosa and related conditions<br>Other necrotising vasculopathies<br>Systemic Lupus Erythematosus<br>Dermatopolymyositis<br>Systemic sclerosis<br>Other systemic involvement of connective tissue<br>Ankylosing spondylitis	These were patients diagnosed with rheumatoid arthritis, systemic lupus erythematosis, systematic sclerosis and mixed connective tissue disease.
Haematological Malignancy	C81 to C96	Malignant neoplasms of lymphoid, haematopoietic and related tissues.	These included patients with any haematological malignancy (lymphoma, myeloma and leukaemia).
Non-Haematological Malignancy	C00 to C75<br><br><br>C76 to C80	Malignant neoplasms, stated or presumed to be primary, of specified sites, except of lymphoid, haematopoietic and related tissues.<br>Malignant neoplasms of ill-defined, secondary and unspecified sites.	These included patients with any non-haematological malignancy.

Diagnosis	SMR01 Definition		Case note review definition
	**ICD-10 Code (2007 Version)**	**ICD-10 Definition**	
	C97	Malignant neoplasms of independent (primary) multiple sites.	
Chronic Liver disease	B18<br>K70.0<br>K70.2-70.9<br><br>K70.0-70.3<br><br>K71.3-71.5<br>K71.7<br><br>K71.1<br>K72.9<br>K76.5<br>K72.1<br>K73<br>K74<br>K76.6<br>K76.7<br>K76.0<br>K76.2-76.4<br><br>K76.8<br>K76.9<br>Z94.4<br>I85.0<br>I85.9<br>I86.4<br>I98.2	Chronic viral hepatitis<br>Alcoholic Fatty Liver<br>Alcoholic liver disease (excluding alcoholic hepatitis K70.1)<br>Alcoholic:- fatty liver; hepatitis; fibrosis and sclerosis of the liver; cirrohisis of the liver<br>Toxic liver disease with chronic hepatitis<br>Toxic Liver disease with fibrosis and cirrhosis of the liver<br>Toxic liver disease with hepatic failure<br>Hepatic failure unspecified<br>Hepatic veno-occlusive disease<br>Chronic hepatic failure<br>Chronic hepatitis, not elsewhere classified<br>Fibrosis and cirrhosis of the liver<br>Portal Hypertension<br>Hepatorenal syndrome<br>Fatty (change of) liver, not elsewhere classified<br>Central haemorrhagic necrosis of liver; infarction of the liver; peliosis hepatitis<br>Other specified diseases of the liver<br>Liver disease unspecified<br>Liver transplant status<br>Oesophageal varices with bleeding<br>Oesophageal varices without bleeding<br>Gastric varices<br>Oesophageal varices in diseases classified elsewhere	Patients were said to have chronic liver disease if there was a clear diagnosis of cirrhosis or chronic active hepatitis proven by imaging, liver biopsy or by a hepatologist.

Diagnosis	SMR01 Definition		Case note review definition
	ICD-10 Code (2007 Version)	ICD-10 Definition	
Smoking status	Z72.0<br>F17.2<br>Z71.6	Tobacco use – personal<br>Tobacco dependence<br>Tobacco abuse counselling	Patients were grouped according to whether they were non-smokers, ex-smokers, current smokers or smoking status unknown.

Note: For all diagnoses except ischaemic heart disease and hypertension, SMR01 data were obtained for the five years prior to the index date, *excluding* the admission at index date. For ischaemic heart disease and hypertension, SMR01 data for the year 2003 were included to match CNR time periods.
