# Supplementary material for: Approaches to ascertaining comorbidity information: validation of routine hospital episode data with clinician-based case note review
Source: BMC Res Notes. 2014 Apr 21;7:253. doi: 10.1186/1756-0500-7-253 (PMC4022331; doi:10.1186/1756-0500-7-253)
Supplement: Additional file 2 — Results of subgroup analysis. [file 1756-0500-7-253-S2.pdf]

Additional file 2: Results of subgroup analysis

Comorbidity	under 75 yrs									75 yrs and over								
	Prevalence				Kappa	Sensitivity	Specificity	PPV	NPV	Prevalence				Kappa	Sensitivity	Specificity	PPV	NPV
	SMR01		CNR							SMR01		CNR						
	n	%	*n*	%		%	%	%	%	*n*	%	*n*	%		%	%	%	%
Ischaemic Heart Disease	383	32.5	422	35.8	0.71	77.0	92.3	84.9	87.8	763	37.4	855	41.9	0.58	70.8	86.7	79.3	80.4
Hypertension	366	31.0	697	59.1	0.28	43.6	87.2	83.1	51.7	562	27.6	1018	49.9	0.28	41.7	86.5	75.4	59.8
Cerebrovascular Disease	92	7.8	71	6.0	0.82	95.8	97.8	73.9	99.7	190	9.3	177	8.7	0.79	83.6	97.7	77.9	98.4
Peripheral Vascular Disease	93	7.9	122	10.3	0.37	37.7	95.6	49.5	93.0	157	7.7	257	12.6	0.40	36.6	96.5	59.9	91.3
Congestive Cardiac Failure	151	12.8	155	13.1	0.53	58.7	94.1	60.3	93.8	360	17.7	391	19.2	0.41	49.9	90.0	54.2	88.3
Diabetes Mellitus	247	20.9	405	34.3	0.63	58.3	98.6	95.5	81.9	254	12.5	400	19.6	0.66	58.5	98.8	92.1	90.7
Dementia	8	0.7	14	1.2	0.36	28.6	99.7	50.0	99.1	83	4.1	173	8.5	0.37	30.1	98.3	62.7	93.8
Chronic Obstructive Pulmonary Disease	90	7.6	95	8.1	0.55	56.8	96.7	60.0	96.2	165	8.1	188	9.2	0.49	50.5	96.2	57.6	95.0
Connective Tissue Disease	69	5.8	69	5.8	0.65	66.7	97.9	66.7	97.9	71	3.5	85	4.2	0.45	43.5	98.3	52.1	97.6
Haematological Malignancy	20	1.7	31	2.6	0.70	58.1	99.8	90.0	98.9	19	0.9	47	2.3	0.48	34.0	99.8	84.2	98.5
Non-haematological Malignancy	95	8.1	137	11.6	0.58	52.6	97.8	75.8	94.0	205	10.1	317	15.5	0.48	44.8	96.3	69.3	90.5
Chronic Liver Disease	23	1.9	23	1.9	0.56	56.5	99.1	56.5	99.1	14	0.7	10	0.5	0.41	50.0	99.6	35.7	99.8
Current or ex-smoker	12	1.0	647	54.8	0.00	1.2	99.2	66.7	45.3	9	0.4	917	45.0	0.01	1.0	100.0	100.0	55.3

Comorbidity	Male									Female								
	Prevalence				Kappa	Sensitivity	Specificity	PPV	NPV	Prevalence				Kappa	Sensitivity	Specificity	PPV	NPV
	SMR01		CNR							SMR01		CNR						
	n	**%**	***n***	**%**		**%**	**%**	**%**	**%**	***n***	**%**	***n***	**%**		**%**	**%**	**%**	**%**
Ischaemic Heart Disease	571	40.4	627	44.3	0.64	75.4	87.5	82.8	81.7	575	31.9	650	36.0	0.62	70.3	89.8	79.5	84.3
Hypertension	398	28.1	744	52.6	0.27	41.3	86.4	77.1	57.0	530	29.4	971	53.8	0.29	43.4	86.9	79.4	56.9
Cerebrovascular Disease	144	10.2	134	9.5	0.82	86.6	97.8	80.6	98.6	138	7.6	114	6.3	0.78	87.7	97.8	72.5	99.2
Peripheral Vascular Disease	129	9.1	193	13.6	0.39	37.8	95.4	56.6	90.7	121	6.7	186	10.3	0.39	36.0	96.7	55.4	92.9
Congestive Cardiac Failure	264	18.7	283	20.0	0.46	54.4	90.3	58.3	88.8	247	13.7	263	14.6	0.44	50.2	92.5	53.4	91.6
Diabetes Mellitus	224	15.8	371	26.2	0.62	55.8	98.4	92.4	86.2	277	15.3	434	24.0	0.68	60.6	99.0	94.9	88.8
Dementia	26	1.8	55	3.9	0.30	23.6	99.0	50.0	97.0	65	3.6	132	7.3	0.41	32.6	98.7	66.2	94.9
Chronic Obstructive Pulmonary Disease	126	8.9	130	9.2	0.49	53.1	95.6	54.8	95.3	129	7.1	153	8.5	0.53	52.3	97.0	62.0	95.6
Connective Tissue Disease	42	3.0	39	2.8	0.53	56.4	98.5	52.4	98.8	98	5.4	115	6.4	0.55	53.0	97.8	62.2	96.8
Haematological Malignancy	18	1.3	36	2.5	0.55	41.7	99.8	83.3	98.5	21	1.2	42	2.3	0.60	45.2	99.9	90.5	98.7
Non-haematological Malignancy	163	11.5	222	15.7	0.54	52.3	96.1	71.2	91.5	137	7.6	232	12.9	0.48	42.2	97.5	71.5	92.0
Chronic Liver Disease	19	1.3	16	1.1	0.51	56.3	99.3	47.4	99.5	18	1.0	17	0.9	0.51	52.9	99.5	50.0	99.6
Current or ex-smoker	11	0.8	883	62.4	0.01	1.1	99.8	90.9	37.8	10	0.6	681	37.7	0.01	1.0	99.7	70.0	62.5

Comorbidity	CKD stage 3a and 3b									CKD stages 4 and 5								
	Prevalence SMR01 *n*	Prevalence SMR01 %	Prevalence CNR *n*	Prevalence CNR %	Kappa	Sensitivity %	Specificity %	PPV %	NPV %	Prevalence SMR01 *n*	Prevalence SMR01 %	Prevalence CNR *n*	Prevalence CNR %	Kappa	Sensitivity %	Specificity %	PPV %	NPV %
Ischaemic Heart Disease	752	34.9	873	40.5	0.63	71.7	90.2	83.2	82.4	394	37.1	404	38.0	0.62	75.2	86.3	77.2	85.1
Hypertension	564	26.2	1139	52.8	0.29	40.1	89.5	81.0	57.2	364	34.2	576	54.2	0.27	47.0	80.9	74.5	56.4
Cerebrovascular Disease	173	8.0	151	7.0	0.77	84.1	97.7	73.4	98.8	109	10.3	97	9.1	0.85	91.8	97.9	81.7	99.2
Peripheral Vascular Disease	160	7.4	251	11.6	0.40	37.1	96.5	58.1	92.1	90	8.5	128	12.0	0.37	36.7	95.4	52.2	91.7
Congestive Cardiac Failure	308	14.3	362	16.8	0.46	50.6	93.0	59.4	90.3	203	19.1	184	17.3	0.43	56.0	88.6	50.7	90.6
Diabetes Mellitus	324	15.0	542	25.1	0.63	55.7	98.6	93.2	86.9	177	16.7	263	24.7	0.70	63.9	98.9	94.9	89.3
Dementia	45	2.1	105	4.9	0.35	26.7	99.2	62.2	96.4	46	4.3	82	7.7	0.40	34.1	98.2	60.9	94.7
Chronic Obstructive Pulmonary Disease	172	8.0	208	9.6	0.53	52.4	96.8	63.4	95.0	83	7.8	75	7.1	0.47	53.3	95.6	48.2	96.4
Connective Tissue Disease	98	4.5	109	5.1	0.54	53.2	98.0	59.2	97.5	42	4.0	45	4.2	0.56	55.6	98.3	59.5	98.0
Haematological Malignancy	27	1.3	55	2.6	0.58	43.6	99.9	88.9	98.5	12	1.1	23	2.2	0.56	43.5	99.8	83.3	98.8
Non-haematological Malignancy	218	10.1	346	16.0	0.54	48.6	97.2	77.1	90.8	82	7.7	108	10.2	0.43	42.6	96.2	56.1	93.7
Chronic Liver Disease	26	1.2	20	0.9	0.43	50.0	99.3	38.5	99.5	11	1.0	13	1.2	0.66	61.5	99.7	72.7	99.5
Current or ex-smoker	14	0.6	1114	51.7	0.01	1.1	99.8	85.7	48.6	7	0.7	450	42.3	0.01	1.1	99.7	71.4	57.9

	<3 comorbidities										3 or more comorbidities									
	Prevalence										Prevalence									
**Comorbidity**	**SMR01**		**CNR**		**Kappa**	**Sensitivity**	**Specificity**	**PPV**	**NPV**		**SMR01**		**CNR**		**Kappa**	**Sensitivity**	**Specificity**	**PPV**	**NPV**
	n	**%**	***n***	**%**		**%**	**%**	**%**	**%**		***n***	**%**	***n***	**%**		**%**	**%**	**%**	**%**
Ischaemic Heart Disease	563	25.4	581	26.3	0.60	69.4	90.2	71.6	89.2		583	58.0	696	69.2	0.52	75.7	81.9	90.4	60.0
Hypertension	504	22.8	999	45.1	0.25	35.6	87.8	70.6	62.4		424	42.1	716	71.2	0.26	52.0	82.1	87.7	40.9
Cerebrovascular Disease	133	6.0	108	4.9	0.77	87.0	98.1	70.7	99.3		149	14.8	140	13.9	0.82	87.1	96.9	81.9	97.9
Peripheral Vascular Disease	97	4.4	100	4.5	0.27	30.0	96.8	30.9	96.7		153	15.2	279	27.7	0.39	39.4	94.1	71.9	80.2
Congestive Cardiac Failure	245	11.1	210	9.5	0.35	44.8	92.5	38.4	94.1		266	26.4	336	33.4	0.49	57.1	89.0	72.2	80.5
Diabetes Mellitus	182	8.2	322	14.6	0.59	49.7	98.8	87.9	92.0		319	31.7	483	48.0	0.63	64.2	98.3	97.2	74.8
Dementia	46	2.1	74	3.3	0.35	29.7	98.9	47.8	97.6		45	4.5	113	11.2	0.39	30.1	98.8	75.6	91.8
Chronic Obstructive Pulmonary Disease	120	5.4	97	4.4	0.41	49.5	96.6	40.0	97.7		135	13.4	186	18.5	0.56	54.3	95.9	74.8	90.2
Connective Tissue Disease	79	3.6	79	3.6	0.45	46.8	98.0	46.8	98.0		61	6.1	75	7.5	0.65	61.3	98.4	75.4	96.9
Haematological Malignancy	31	1.4	52	2.3	0.62	50.0	99.8	83.9	98.8		8	0.8	26	2.6	0.46	30.8	100.0	100.0	98.2
Non-haematological Malignancy	183	8.3	225	10.2	0.52	51.1	96.6	62.8	94.6		117	11.6	229	22.8	0.49	43.2	97.7	84.6	85.4
Chronic Liver Disease	13	0.6	14	0.6	0.59	57.1	99.8	61.5	99.7		10	1.0	19	1.9	0.55	42.1	99.8	80.0	98.9
Current or ex-smoker	11	0.5	988	44.6	0.00	0.7	99.7	63.6	55.4		10	1.0	576	57.3	0.01	1.7	100.0	100.0	43.2

	No IHD										IHD									
	**Prevalence**										**Prevalence**									
**Comorbidity**	**SMR01**		**CNR**		**Kappa**	**Sensitivity**	**Specificity**	**PPV**	**NPV**		**SMR01**		**CNR**		**Kappa**	**Sensitivity**	**Specificity**	**PPV**	**NPV**
	n	**%**	***n***	**%**		**%**	**%**	**%**	**%**		***n***	**%**	***n***	**%**		**%**	**%**	**%**	**%**
Hypertension	504	26.0	1096	56.4	0.26	38.1	89.8	82.9	52.9		424	33.2	619	48.5	0.33	50.1	82.7	73.1	63.8
Cerebrovascular Disease	144	7.4	123	6.3	0.80	88.6	98.1	75.7	99.2		138	10.8	125	9.8	0.79	85.6	97.3	77.5	98.4
Peripheral Vascular Disease	125	6.4	188	9.7	0.40	37.2	96.9	56.0	93.5		125	9.8	191	15.0	0.37	36.6	94.9	56.0	89.5
Congestive Cardiac Failure	178	9.2	194	10.0	0.44	46.9	95.0	51.1	94.2		333	26.1	352	27.6	0.41	55.4	85.1	58.6	83.4
Diabetes Mellitus	256	13.2	454	23.4	0.61	52.6	98.9	93.4	87.2		245	19.2	351	27.5	0.71	65.8	98.5	94.3	88.4
Dementia	62	3.2	116	6.0	0.40	32.8	98.7	61.3	95.9		29	2.3	71	5.6	0.34	25.4	99.1	62.1	95.8
Chronic Obstructive Pulmonary Disease	127	6.5	127	6.5	0.49	52.8	96.7	52.8	96.7		128	10.0	156	12.2	0.53	52.6	95.9	64.1	93.6
Connective Tissue Disease	85	4.4	99	5.1	0.54	52.5	98.2	61.2	97.5		55	4.3	55	4.3	0.54	56.4	98.0	56.4	98.0
Haematological Malignancy	31	1.6	61	3.1	0.60	45.9	99.8	90.3	98.3		8	0.6	17	1.3	0.48	35.3	99.8	75.0	99.1
Non-haematological Malignancy	192	9.9	286	14.7	0.54	50.0	97.0	74.5	91.8		108	8.5	168	13.2	0.46	42.3	96.7	65.7	91.7
Chronic Liver Disease	17	0.9	23	1.2	0.55	47.8	99.7	64.7	99.4		6	0.5	10	0.8	0.62	50.0	99.9	83.3	99.6
Current or ex-smoker	10	0.5	835	43.0	0.01	0.8	99.7	70.0	57.1		11	0.9	729	57.1	0.01	1.4	99.8	90.9	43.2

Comorbidity	No malignancy									Malignancy								
	Prevalence				Kappa	Sensitivity	Specificity	PPV	NPV	Prevalence				Kappa	Sensitivity	Specificity	PPV	NPV
	SMR01		CNR							SMR01		CNR						
	n	%	*n*	%		%	%	%	%	*n*	%	*n*	%		%	%	%	%
Ischaemic Heart Disease	977	36.3	1094	40.6	0.63	73.3	89.1	82.1	83.0	169	32.1	183	34.8	0.59	69.9	88.0	75.7	84.6
Hypertension	796	29.6	1475	54.8	0.28	42.7	86.4	79.1	55.5	132	25.1	240	45.6	0.30	40.8	88.1	74.2	64.0
Cerebrovascular Disease	250	9.3	218	8.1	0.81	89.0	97.7	77.6	99.0	32	6.1	30	5.7	0.69	73.3	98.0	68.8	98.4
Peripheral Vascular Disease	215	8.0	330	12.3	0.38	36.7	96.0	56.3	91.6	35	6.7	49	9.3	0.41	38.8	96.6	54.3	93.9
Congestive Cardiac Failure	446	16.6	479	17.8	0.45	52.2	91.1	56.1	89.8	65	12.4	67	12.7	0.48	53.7	93.7	55.4	93.3
Diabetes Mellitus	441	16.4	705	26.2	0.67	59.6	98.9	95.2	87.3	60	11.4	100	19.0	0.56	50.0	97.7	83.3	89.3
Dementia	80	3.0	160	5.9	0.37	29.4	98.7	58.8	95.7	11	2.1	27	5.1	0.46	33.3	99.6	81.8	96.5
Chronic Obstructive Pulmonary Disease	209	7.8	235	8.7	0.50	51.5	96.4	57.9	95.4	46	8.7	48	9.1	0.56	58.3	96.2	60.9	95.8
Connective Tissue Disease	119	4.4	130	4.8	0.51	50.8	97.9	55.5	97.5	21	4.0	24	4.6	0.74	70.8	99.2	81.0	98.6
Chronic Liver Disease	19	0.7	30	1.1	0.61	50.0	99.8	78.9	99.4	<5	<0.9	<5	<0.9	0.28	33.3	99.4	25.0	99.6
Current or ex-smoker	21	0.8	1298	48.2	0.01	1.3	99.7	81.0	52.1	0	0.0	266	50.6	0.00	0.0	100.0	-	49.4

Comorbidity	UR 1&2									UR 3-6								
	Prevalence									Prevalence								
	SMR01		CNR		Kappa	Sensitivity	Specificity	PPV	NPV	SMR01		CNR		Kappa	Sensitivity	Specificity	PPV	NPV
	n	%	*n*	%		%	%	%	%	*n*	%	*n*	%		%	%	%	%
Ischaemic Heart Disease	665	37.4	738	41.5	0.63	73.6	88.3	81.7	82.5	469	33.5	524	37.4	0.63	71.9	89.5	80.4	84.2
Hypertension	524	29.5	966	54.3	0.30	43.6	87.3	80.3	56.6	393	28.1	727	51.9	0.27	41.1	86.1	76.1	57.5
Cerebrovascular Disease	178	10.0	158	8.9	0.79	86.1	97.4	76.4	98.6	101	7.2	88	6.3	0.81	88.6	98.2	77.2	99.2
Peripheral Vascular Disease	147	8.3	232	13.0	0.40	37.5	96.1	59.2	91.1	96	6.9	143	10.2	0.36	34.3	96.3	51.0	92.8
Congestive Cardiac Failure	286	16.1	315	17.7	0.46	52.4	91.7	57.7	90.0	219	15.6	226	16.1	0.43	51.3	91.2	53.0	90.7
Diabetes Mellitus	281	15.8	448	25.2	0.66	59.2	98.8	94.3	87.8	214	15.3	348	24.8	0.64	57.2	98.6	93.0	87.4
Dementia	49	2.8	121	6.8	0.35	26.4	99.0	65.3	94.9	41	2.9	64	4.6	0.44	37.5	98.7	58.5	97.1
Chronic Obstructive Pulmonary Disease	163	9.2	188	10.6	0.52	53.2	96.0	61.3	94.6	90	6.4	93	6.6	0.49	51.6	96.8	53.3	96.6
Connective Tissue Disease	82	4.6	81	4.6	0.54	56.8	97.9	56.1	97.9	56	4.0	71	5.1	0.53	49.3	98.4	62.5	97.3
Haematological Malignancy	19	1.1	39	2.2	0.58	43.6	99.9	89.5	98.8	20	1.4	39	2.8	0.57	43.6	99.8	85.0	98.4
Non-haematological Malignancy	169	9.5	263	14.8	0.54	48.7	97.3	75.7	91.6	127	9.1	184	13.1	0.48	45.7	96.5	66.1	92.2
Chronic Liver Disease	14	0.8	19	1.1	0.54	47.4	99.7	64.3	99.4	9	0.6	14	1.0	0.61	50.0	99.9	77.8	99.5
Current or ex-smoker	11	0.6	899	50.5	0.01	0.9	99.7	72.7	49.6	10	0.7	648	46.3	0.01	1.4	99.9	90.0	54.1

	SIMD 1-3									SIMD 4&5								
	Prevalence									Prevalence								
**Comorbidity**	**SMR01**		**CNR**		**Kappa**	**Sensitivity**	**Specificity**	**PPV**	**NPV**	**SMR01**		**CNR**		**Kappa**	**Sensitivity**	**Specificity**	**PPV**	**NPV**
	n	**%**	***n***	**%**		**%**	**%**	**%**	**%**	***n***	**%**	***n***	**%**		**%**	**%**	**%**	**%**
Ischaemic Heart Disease	584	36.9	633	40.0	0.62	73.6	87.6	79.8	83.3	550	34.5	629	39.4	0.64	72.2	90.1	82.5	83.3
Hypertension	481	30.4	849	53.6	0.30	44.8	86.3	79.0	57.5	436	27.3	844	52.9	0.27	40.3	87.2	78.0	56.6
Cerebrovascular Disease	143	9.0	119	7.5	0.78	87.4	97.3	72.7	99.0	136	8.5	127	8.0	0.82	86.6	98.2	80.9	98.8
Peripheral Vascular Disease	109	6.9	191	12.1	0.37	33.5	96.8	58.7	91.4	134	8.4	184	11.5	0.39	39.1	95.6	53.7	92.3
Congestive Cardiac Failure	255	16.1	267	16.9	0.45	53.2	91.4	55.7	90.6	250	15.7	274	17.2	0.44	50.7	91.6	55.6	90.0
Diabetes Mellitus	268	16.9	424	26.8	0.65	59.2	98.5	93.7	86.9	227	14.2	372	23.3	0.65	57.3	98.9	93.8	88.4
Dementia	41	2.6	82	5.2	0.39	30.5	98.9	61.0	96.3	49	3.1	103	6.5	0.38	30.1	98.8	63.3	95.3
Chronic Obstructive Pulmonary Disease	145	9.2	159	10.0	0.50	52.2	95.6	57.2	94.7	108	6.8	122	7.6	0.53	53.3	97.1	60.2	96.2
Connective Tissue Disease	76	4.8	78	4.9	0.56	57.7	97.9	59.2	97.8	62	3.9	74	4.6	0.51	48.6	98.3	58.1	97.5
Haematological Malignancy	14	0.9	32	2.0	0.52	37.5	99.9	85.7	98.7	25	1.6	46	2.9	0.61	47.8	99.8	88.0	98.5
Non-haematological Malignancy	141	8.9	220	13.9	0.54	48.6	97.5	75.9	92.2	155	9.7	227	14.2	0.49	46.3	96.3	67.7	91.5
Chronic Liver Disease	14	0.9	19	1.2	0.60	52.6	99.7	71.4	99.4	9	0.6	14	0.9	0.52	42.9	99.8	66.7	99.5
Current or ex-smoker	15	0.9	809	51.1	0.01	1.6	99.7	86.7	49.3	6	0.4	738	46.2	0.00	0.5	99.8	66.7	53.8
